# Methylphenidate-associated brain structural changes and neuroimaging predictors of treatment response in youth with ADHD: a systematic review

**DOI:** 10.1016/j.nicl.2026.104085

**Published:** 2026-11-03

**Authors:** Liesbeth Reneman, Zarah van der Pal, Daniëlle de Smidt, Shandra Bipat

**Affiliations:** Department of Radiology & Nuclear Medicine, Amsterdam UMC location University of Amsterdam, Amsterdam, the Netherlands

**Keywords:** Attention-deficit/hyperactivity disorder (ADHD), Methylphenidate, Brain development, Magnetic resonance imaging (MRI)

## Abstract

**Background:**

Methylphenidate (MPH) is a psychostimulant widely prescribed to children with attention-deficit/hyperactivity disorder (ADHD). Although its clinical efficacy is well established, its lasting effects on the developing brain remain unclear.

**Objective:**

This systematic review aimed to examine associations between long-term MPH treatment and brain structure in children and adolescents with ADHD using structural magnetic resonance imaging (sMRI) and diffusion tensor imaging (DTI), distinguishing longitudinal treatment-effect studies from cross-sectional medication-history comparisons and baseline neuroimaging predictors of treatment response.

**Methods:**

A systematic search was conducted in MEDLINE and Embase databases. Studies were included if they investigated structural brain changes (gray matter volume, white matter integrity and cortical thickness) after at least four weeks of MPH treatment in children with ADHD. Data extraction and quality assessment were performed using the NIH quality assessment tool.

**Results:**

Eight studies met the inclusion criteria: three longitudinal studies using repeated MRI to assess within-subject treatment effects, two cross-sectional comparisons based on medication history, and three studies using baseline neuroimaging to predict subsequent treatment response. Greater putamen volume was associated with medication status ad volume with better treatment response in the cross-sectional and predictive analyses, while cortical effects were less consistent and mainly age-dependent in frontal areas. White matter findings were subtle and inconsistent across studies, and cortical thickness evidence—limited to one study—suggests short-term, possibly transient effects in children.

**Conclusion:**

MPH treatment is associated l with age-dependent differences in brain structure in children and adolescents with ADHD; however, the limited number of included studies varied substantially in design, (cross-sectional and predictive studies alongside longitudinal treatment-effect studies), methodology, and reported outcomes, making firm causal conclusions difficult. Future research should prioritize larger, standardized, long-term longitudinal studies that account for developmental factors.

## Highlights

• There is limited evidence on long-term methylphenidate effects on the developing brain.
• Methylphenidate seems linked to increased subcortical volume, especially putamen.
• Frontal cortical effects are more variable and white matter findings are subtle and inconsistent.
• Across all outcomes, age appeared to be a key moderating factor.

## 1 Introduction

Attention-deficit/hyperactivity disorder (ADHD) is the most commonly diagnosed neurobehavioral condition in childhood population-prevalence of 5.3% (Polanczyk et al., 2007). The DSM-5 classifies attention-deficit/hyperactivity disorder (ADHD) into three presentations: predominantly inattentive, predominantly hyperactive-impulsive, and combined presentation (Magnus et al., 2023). ADHD is therefore considered a heterogeneous disorder and is associated with a substantial financial burden, increased family stress, and adverse academic outcomes (Biederman, 2005). Symptoms typically emerge in early childhood and include inattention, difficulty sustaining concentration, challenges in completing tasks, and forgetfulness or frequently losing items (Magnus et al., 2023). Not only is it a prevalent condition, but increasingly greater numbers of children and young adolescents are exposed to pharmacological treatments, many of whom likely do not even meet the criteria for ADHD (Elder, 2010). In addition, it is estimated that between more than 60% of children diagnosed with ADHD are affected by one or more comorbid conditions, including oppositional defiant disorder, anxiety and mood disorders, sleep disorders, autistic spectrum disorder, tic disorders, and learning disabilities (Gnanavel et al., 2019; Schweren et al., 2013). ADHD is thought to involve alterations in catecholaminergic neurotransmission, particularly affecting dopamine transporters (Hayashi and Iwanami, 2018; Prince, 2008), which may result in reduced dopamine availability in the synaptic cleft (Verghese et al., 2024). Methylphenidate (MPH), the most commonly prescribed treatment for ADHD, inhibits the reuptake of dopamine and norepinephrine, thereby increasing their synaptic availability, primarily in the prefrontal cortex (Verghese et al., 2024). It has been shown to effectively reduce symptoms of inattention, hyperactivity, and impulsivity in up to 80% of affected children and its short-term safety is well-documented; however, its long-term effects remain insufficiently understood.

This gap is particularly concerning given the heightened neuroplasticity of the developing brain, which may render it more vulnerable to pharmacological interference with neurotransmitter systems. Existing evidence, largely derived from studies in healthy male animals, suggests that while stimulant exposure in adults induces only transient neuroadaptive changes, exposure during juvenile stages may result in more persistent or even permanent alterations, a phenomenon described as ‘neurochemical imprinting’ (Andersen and Navalta, 2004). Despite these findings, research in humans on the long-term impact of MPH in children is scarce, prompting increasing concern regarding potential adverse developmental consequences. Accordingly, major regulatory and research bodies have underscored the urgent need for comprehensive investigations into its long-term safety profile in pediatric populations including the US Food and Drug Administration (Center for Drug Evaluation and Research, 2004), the National Institutes of Health (*J. Am. Acad. Child Adolesc. Psychiatry*, 2000) and the European Committee for Medicinal Products for Human Use (Committee for Medicinal Products for Human Use, 2008), and carefully evaluate both its beneficial and potential adverse effects on the brain.

To examine these effects in children with ADHD, magnetic resonance imaging (MRI) is frequently used (Kim et al., 2022; Schweren et al., 2015). Systematic reviews are considered to provide the highest levels of evidence in scientific research, as they synthesize findings across multiple studies. Their strength lies in their comprehensive, transparent, and reproducible methodology, which enhances the reliability and precision of their conclusions. In addition, systematic reviews play a key role in informing clinical guidelines and evidence-based practice (Cook et al., 1997), as they summarize all relevant studies, making the available evidence more accessible (Gopalakrishnan and Ganeshkumar, 2013). This systematic review aimed to compare studies investigating the long-term effects of MPH treatment on structural brain development in children with ADHD. The primary outcomes included gray matter volume, white matter integrity—measured by fractional anisotropy (FA) and mean diffusivity (MD)—and cortical thickness. We focus specifically on structural MRI to assess more stable, anatomy-based brain changes associated with MPH treatment, as functional MRI captures more transient, state-dependent neural activity that is less directly comparable across studies.

## 2 Materials and methods

This review followed the Preferred Reporting Items for Systematic Reviews and Meta-Analysis (PRISMA) guidelines, which are widely recognized as international standards for systematic review methodology (Page et al., 2021). PRISMA enhances the transparency of the search and selection process and ensures that the results are reproducible (Liberati et al., 2009).

### 2.1 Search strategy and selection and inclusion of papers

#### 2.1.1 Search strategy

The search was conducted until 1 September 2025 using two databases, MEDLINE and EMBASE, which increased the likelihood of identifying relevant studies compared to relying on a single database. From 1 September 2025 until April 2026, databases were checked on additional papers. The search terms used in MEDLINE were attention deficit disorder with hyperactivity AND methylphenidate AND magnetic resonance imaging AND brain. The terms used in EMBASE were ‘attention deficit disorder with hyperactivity’/exp. AND ‘methylphenidate’/exp. AND ‘magnetic resonance imaging’/exp. AND ‘brain’/exp. (see details in Supplement 1). No language restriction was performed.

#### 2.1.2 Selection and inclusion of relevant articles

The selection of potential relevant studies was performed by DS. Titles and abstracts were screened to identify studies with potential relevance. Duplicates, letters, comments, editorials, conference abstracts, other diseases, reviews, studies involving only adults (≥18 years of age) or healthy volunteers, and animal studies were not considered as relevant and were excluded. In case of doubt, the article was checked by SB, a clinical epidemiologist with 20 years of experience in radiology.

The articles identified as potentially relevant were subsequently assessed in full text to determine whether they met the inclusion criteria listed below.

1. A study population of at least 10 individuals treated with MPH
2. Treatment with MPH (defined as at least 4 weeks)
3. Reported outcomes related to gray matter, white matter (measured by fractional anisotropy (FA) and mean diffusivity (MD), and/or cortical thickness

This was independently done by DS and LR (a neuroradiologist with >20 years of experience on MPH and MRI) independently and in case of disagreement, was discussed with SB.

To identify additional papers, the reference lists of the included papers were checked and forward citations of included papers were also checked (in Pubmed) to see who has cited the included papers.

### 2.2 Data extraction on study, patients, medication and MRI characteristics

All articles that met the inclusion criteria were further analyzed using a standardized case record form This part of the data extraction was performed by DS and double checked by SB.

#### 2.2.1 Study characteristics

##### 2.2.1.1 Study design characteristics

A subset of items was used to evaluate the characteristics of the study design: 1) study type; 2) study design; 3) setting initiation institute (department, institution, and country of the first author); 4) period of recruitment; 5) kind of group division; 6) study approval mentioned; 7) source of funding; and 8) whether there was a role of the funders in the study.

#### 2.2.2 Patient characteristics

For the purpose of comparing the included studies, patient characteristics were systematically analyzed according to the following items: 1) whether a consecutive or random sample of individuals was enrolled; 2) whether inclusion/exclusion criteria were specified; 3) number of individuals included in the study; 4) number of individuals included in the analysis; 5) age; 6) sex and/or gender; 7) intelligence quotient; 8) right-handedness; 9) comorbid conditions; 10) treatment naivety; 11) ADHD presentation; and 12) whether the history of head trauma was included.

#### 2.2.3 Medication characteristics

Given the variability in the administration of MPH across studies, this review also included medication characteristics: 1) Duration of MPH treatment and 2) dose of MPH. This review included only MPH studies to ensure comparability, as MPH (a dopamine reuptake inhibitor) differs fundamentally from other stimulants like amphetamines, which act mainly as dopamine releasers.

#### 2.2.4 MRI characteristics and follow-up procedure

To gain insight into the effects of MPH on brain structure as assessed using MRI, the following items were used: 1) type of MRI; 2) MRI strength; 3) number of channels in the head coil; 4) MRI parameters; 5) type of analysis; 6) number of follow-up assessments; 7) total follow-up time; 8) report of dropouts; and 9) sufficient description of follow-up procedure.

### 2.3 Data extraction on outcomes

This part of the data extraction was also performed by DS and double checked by SB and LR.

The outcomes extracted were 1) gray matter volume, 2) white matter integrity (FA, fractional anisotropy; MD, mean diffusivity) and 3) cortical thickness.

### 2.4 Data extraction on methodological quality assessment

This part of the data extraction was also performed by DS and double checked by SB and LR.

The National Institutes of Health quality assessment tool for case series (National Institutes of Health, 2021) was used to evaluate the methodological quality of the included studies. This assessment considered multiple aspects, including the patient population, outcome measures, follow-up procedures, and statistical methods.

To minimize selection bias, the following data were collected:

Q1: Was the study question or objective clearly stated.

Q2: Was the study population clearly and fully described, including the case definition?

Q3: Were the cases consecutive?;

Q4: Were the subjects comparable?

Q5: Was the intervention described clearly?;

Q6: Were the outcome measures clearly defined, valid, reliable, and consistently implemented across all study participants?

Q7: Was the follow-up duration adequate?;

Q8: Were the statistical methods well described?

Q9: Were the results well described?

The answer options for the abovementioned questions (Q1-Q9) were ‘yes’, ‘no’, or ‘cannot determine/not applicable/not reported’. To evaluate the overall quality, an additional rating was assigned, categorized as ‘good’, ‘fair’, or ‘poor’*.* Overall quality was ‘good’ if 7–9 questions were answered with ‘yes’, ‘fair’ if 4–6 questions were answered with ‘yes’, and ‘poor’ if less than 4 questions were answered with ‘yes’.

For the consistency, National Institutes of Health quality assessment tool of controlled intervention studies (National Institutes of Health, 2021) was used to evaluate the methodological quality of the randomized controlled trials (RCT). This assessment considered all aspects of RCT including randomization, allocation concealment, blinding, attrition (follow-up), or confounding,

The following data were collected:

Q1. Was the study described as randomized, a randomized trial, a randomized clinical trial, or an RCT?

Q2. Was the method of randomization adequate (i.e., use of randomly generated assignment)?

Q3. Was the treatment allocation concealed (so that assignments could not be predicted)?

Q4. Were study participants and providers blinded to treatment group assignment?

Q5. Were the people assessing the outcomes blinded to the participants' group assignments?

Q6. Were the groups similar at baseline on important characteristics that could affect outcomes (e.g., demographics, risk factors, co-morbid conditions)?

Q7. Was the overall drop-out rate from the study at endpoint 20% or lower of the number allocated to treatment?

Q8. Was the differential drop-out rate (between treatment groups) at endpoint 15 percentage points or lower?

Q9. Was there high adherence to the intervention protocols for each treatment group?

Q10. Were other interventions avoided or similar in the groups (e.g., similar background treatments)?

Q11. Were outcomes assessed using valid and reliable measures, implemented consistently across all study participants?

Q12. Did the authors report that the sample size was sufficiently large to be able to detect a difference in the main outcome between groups with at least 80% power?

Q13. Were outcomes reported or subgroups analyzed prespecified (i.e., identified before analyses were conducted)?

Q14. Were all randomized participants analyzed in the group to which they were originally assigned, i.e., did they use an intention-to-treat analysis?

The answer options for the abovementioned questions (Q1-Q14) were ‘yes’, ‘no’, or ‘cannot determine/not applicable/not reported’. To evaluate the overall quality, an additional rating was assigned, categorized as ‘good’, ‘fair’, or ‘poor’*.* Overall quality was ‘good’ if 10–14 questions were answered with ‘yes’, ‘fair’ if 6–9 questions were answered with ‘yes’, and ‘poor’ if less than 6 questions were answered with ‘yes’.

## 3 Results

### 3.1 Search strategy and selection of relevant papers

Following our search strategy, 507 articles were identified (see Supplement 1). After screening titles and abstracts, 27 articles were considered potentially relevant. The full texts of these studies were then evaluated against the inclusion criteria described above: minimum of 10 children (< 18 years) with ADHD included, at least 4-week treatment with MPH, and evaluation of the outcomes of interest. This process resulted in inclusion of eight studies (Park et al., 2013; de Luis-García et al., 2015; Villemonteix et al., 2015; Bouziane et al., 2019; Walhovd et al., 2020; Chang et al., 2021; Chen et al., 2025; Chang et al., 2025), see Fig. 1. No additional papers were found between September 2025 and April 2026. No additional papers were found after checking reference lists and forward citation of the included papers.

**Fig. 1:**
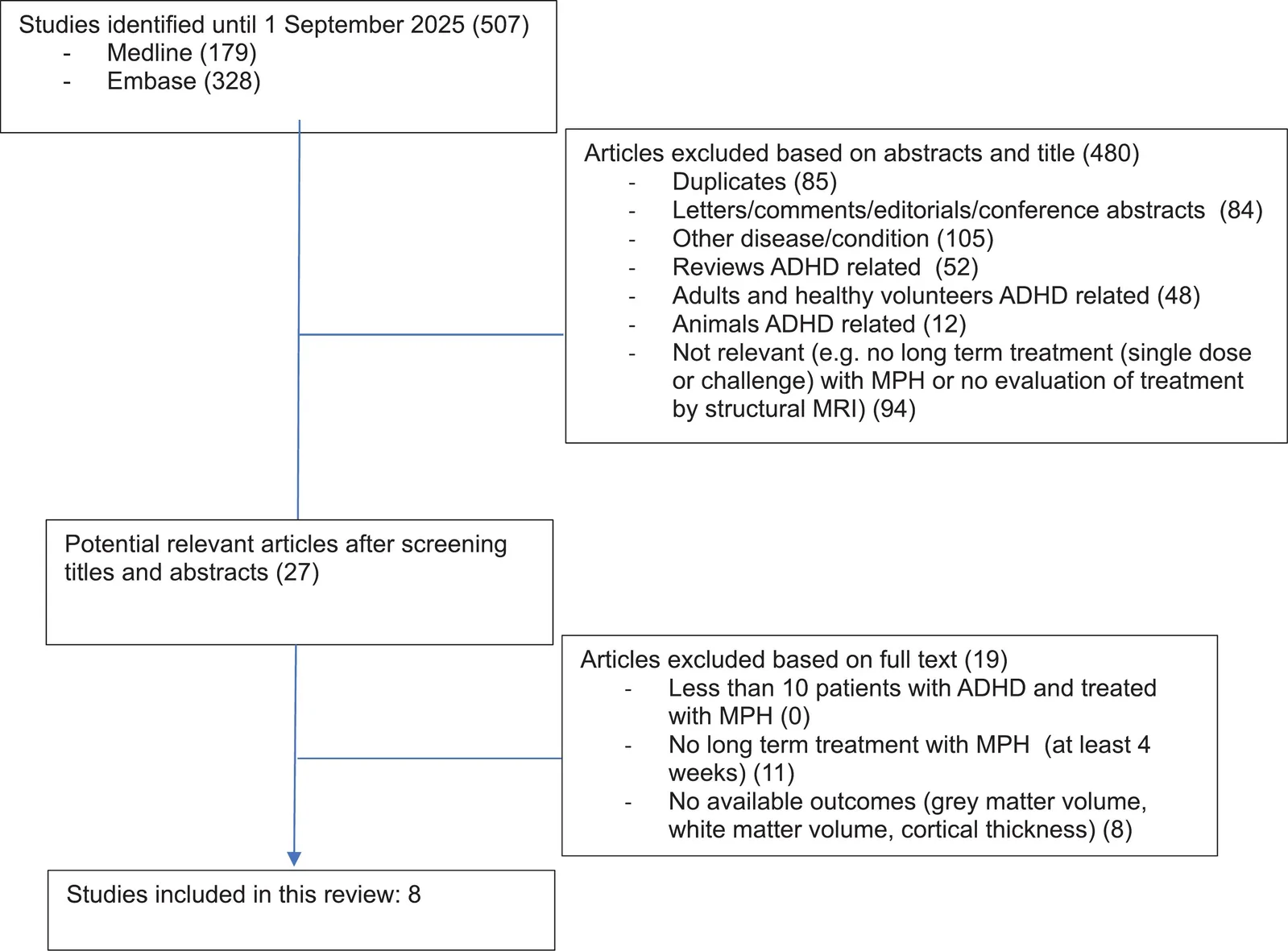
Flowchart describing the results of the search and selection of relevant papers.

### 3.2 Study design, patient, medication and MRI characteristics

#### 3.2.1 Study design characteristics

Table 1 presents the study design characteristics of the included studies. The majority of the included studies were cohort designs, whereas only 1 RCT was included, as described in three publications: Bouziane et al. (2019), Walhovd et al. (2020), and Chen et al. (2025). Almost all studies mentioned their ethical approval, except for the study by de Luis-García et al. (2015). Importantly, all studies disclosed their sources of funding, and nearly all explicitly stated that the funding bodies had no influence on the research process or the outcomes.

**Table 1:** Study design characteristics.

Study	Study type	Design of study	Department and institution, and country of first author	Period recruitment	Kind of group division	Ethical approval mentioned	Source of funding mentioned	Role of funding in study
Park et al. (2013)	Cohort	Single center	The Div. of Child and Adolescent Psychiatry, Dept. of Psychiatry, Seoul National University College of Medicine, Republic of Korea	N/D	Mspl genotype: with/without C allele Dral genotype: with/without T allele	Yes	Yes	N/D
de Luis-García et al. (2015)	Cohort	N/D	Laboratorio de Procesado de Imagen, Universidad de Valladolid, Spain	N/D	Healthy Children ADHD-nT ADHD-uT	N/D	Yes	N/D
Villemonteix et al. (2015)	Cohort	Multicenter	Centre Psychiatrie et Neurosciences, Institut national de la santé et de la recherche médicale, France	N/D	Healthy children Medicated ADHD Never-medicated ADHD	Yes	Yes	No
Bouziane et al. (2019)	RCT	Multicenter	Departments of Radiology and Nuclear Medicine and Biomedical Engineering and Physics, University of Amsterdam, the Netherlands	2011–2015	MPH/placebo	Yes	Yes	No
Walhovd et al. (2020)	RCT	Multicenter	Department of Psychology, Center for Lifespan Changes in Brain and Cognition, and Institute of Physics, Faculty of Mathematics and Natural Sciences, University of Oslo, Oslo, Norway; Departments of Radiology and Nuclear Medicine and Diagnostic Physics, Oslo University Hospital, Oslo, Norway	2011–2015	MPH/placebo	Yes	Yes	N/D
Chang et al. (2021)	Cohort	Single center	Department of Psychiatry, National Taiwan University Hospital, Taiwan	N/D	Good/poor responders	Yes	Yes	No
Chen et al. (2025)	RCT	Multicenter	Department of Radiology and Nuclear Medicine, Amsterdam University Medical Center, the Netherlands	2011–2015	Responders/non-responders) and children/adults	Yes	Yes	No
Chang et al. (2025)	Cohort	Multicenter	Department of Psychiatry, National Taiwan University Hospital and College of Medicine, Taiwan	N/D	MPH/control	Yes	Yes	No

Abbreviations: RCT, Randomized Controlled Trial; N/D, not determined; ADHD, attention-deficit hyperactivity disorder; ADHD-nT, treatment naïve; ADHD-uT, MPH-treatment; MPH, methylphenidate.

#### 3.2.2 Patient characteristics

Table 2 shows the details of patient characteristics. Substantial variations were observed across the studies. First, the number of participants included in the studies ranged from 50 to 180 children, and the number of participants included in analysis varied between 20 and 89. It is important to note that two studies by Chang et al. (2021) and Chang et al. (2025) also included adult participants (≥18 years), because these data could not be separated from the overall group results. Because the total number of included studies was already limited, we decided to retain these studies in our analysis. The inclusion and exclusion criteria also differed considerably across the included studies. For instance, half of the studies excluded female participants (de Luis-García et al., 2015; Bouziane et al., 2019; Walhovd et al., 2020; Chen et al., 2025), whereas others did not (Park et al., 2013; Villemonteix et al., 2015; Chang et al., 2021; Chang et al., 2025). Two studies, de Luis-García et al. (2015) and Villemonteix et al. (2015), restricted their inclusion to right-handed participants only. Most studies excluded participants with comorbid conditions from their analyses. Only the study by Park et al. (2013) included individuals with anxiety or mild oppositional defiant disorder. Finally, all studies selected participants without prior MPH treatment, with the exception of de Luis-García et al. (2015) and Chang et al. (2025).

**Table 2:** Patient characteristics.

Study	Consecutive or random sample enrolled?	In- and exclusion criteria	Number of individuals included	Number of MPH analyzed individuals	Age (mean + SD)	%Male	IQ (Mean + SD)	100% Right-handed	Comorbidities	Treatment naïve	ADHD subtype	Multiple comparison correction
Park et al. (2013)	Yes	Yes	53	GG:25 GC + CC:28 CC: 15 CT + TT: 38	9.05 (0.36) 9.06 (0.40) 8.90 (0.53) 9.12 (0.32)	84.9	108.60 (2.30) 109.29 (2.75) 109.13 (3.36) 108.89 (2.15)	N/D	Anxiety, MDD	Yes	C (36/53) I (14/53 H (3/53)	No
de Luis-García et al. (2015)	Yes	Yes	66	24	8.50 (1.1)	100	103.3 (13.3)	Yes	No	No	N/D	Yes
Villemonteix et al. (2015)	Yes	Yes	77	20	10.4 (1.4)	80	107.4 (13.5)	Yes	No	Yes	Combined	No
Bouziane et al. (2019)	Yes	Yes	50	25	11.4 (0.8)	100	104.8 (21.0)	No	No	Yes	I (14/25) C (11/25)	Yes
Walhovd et al. (2020)	Yes	Yes	50	24	11.4 (0.8)	100	104.8 (21.0)	No	No	Yes	N/D	No
Chang et al. (2021)⁎	Yes	Yes	79	79	17.6 (10.1)	85.7	107.3 (12.4)	No	No	Yes	I (33/63) H (1/63) C (29/63)	Yes
Chen et al. (2025)⁎	Yes	Yes	50	23	11 (1)	100	104.8 (21.0)	No	No	Yes	I (12/23) C (11/23)	No
Chang et al. (2025)⁎	Yes	Yes	180	89	17.59 (8.00)	76.4	109.10 (8.77)	N/D	No	No	N/D	Yes

Abbreviations: N/D, not determined; C, combined type; I, inattentive type; H, hyperactive type.

⁎ At least 10 children analyzed in the study, but results cannot be separated from the overall adult group.

#### 3.2.3 Medication characteristics

Table 3 presents the medication characteristics of the individuals. The reported treatment duration and dosage varied across the included studies. For instance, Villemonteix et al. (2015) reported the longest minimum treatment period, with a minimum of one year, whereas Chang et al. (2021) reported the shortest, with a minimum of 4 weeks (as per our inclusion criteria). The remaining studies reported treatment durations ranging between 8 weeks and 6 months. Chang et al. (2025) did not clearly report the minimum treatment period; however, the average follow-up interval in this study was 4.86 years.

**Table 3:** Medication characteristics.

Study	Duration of MPH treatment	Dose of MPH
Park et al. (2013)	8 weeks	0.35–1.77 mg/kg/day (clinical guidance)
de Luis-García et al. (2015)	At least 6 months	N/D
Villemonteix et al. (2015)	At least 1 year	3× per day: 0.3–0.5 mg/kg
Bouziane et al. (2019)	16 weeks	Clinical guidance
Walhovd et al. (2020)	16 weeks	Clinical guidance
Chang et al. (2021)	At least 1 month	Immediate Release, dose N/D
Chen et al. (2025)	16 weeks	Clinical guidance
Chang et al. (2025)	On average 4.86 years	Varies

Abbreviations: MPH, methylphenidate; N/D, not determined.

Two studies, Park et al. (2013) and Villemonteix et al. (2015), specified the administered dosage. Park et al. reported dosages ranging from 0.35 to 1.77 mg/kg/day, whereas Villemonteix et al. reported a dosage of 0.3–0.5 mg/kg administered three times per day. In other studies, the dosage varied per patient or was not reported. In the ePOD-MPH RCT (Bouziane et al., 2019; Walhovd et al., 2020; Chen et al., 2025), dosage determination was based on Dutch clinical guidelines (clinical response). Additionally, Chang et al. (2021) specified the use of immediate-release MPH but did not provide details on the exact dosage.

#### 3.2.4 MRI characteristics and follow-up procedure

Table 4 summarizes the MRI characteristics and follow-up procedures of the included studies. Four studies used sMRI (Villemonteix et al., 2015; Walhovd et al., 2020; Chang et al., 2021; Chang et al., 2025), while the remaining four employed DTI sequences (Park et al., 2013; de Luis-García et al., 2015; Bouziane et al., 2019; Chen et al., 2025). These imaging modalities differ in the type of information they provide. sMRI generates detailed images of brain structure, including gray and white matter volume and cortical thickness, whereas DTI assesses white matter integrity and connectivity, typically quantified using measures such as fractional anisotropy (FA) and mean diffusivity (MD) (ExpertMRI, 2024).

**Table 4:** MR I maging characteristics and follow up procedures.

Study	Type MRI	Strength	Channel head coil	MRI parameters	Type analysis	Number FU	Total FU time	DO reported?	Was FU procedure sufficiently described?
Park et al. (2013)	DTI	3.0 T	N/R	TR/TE: 10,000/90 ms; FOV: 240 × 240 mm^2^; flip angle: not reported; In-plane voxel size: not reported; slices: 38	VBA	1	8 weeks	No	Yes
de Luis-García et al. (2015)	DTI	1,5 T	N/R	TR/TE: 13,000/85.5 ms; FOV: not reported; flip angle: not reported; In-plane voxel size: 1.015 × 1.015 × 3 mm^3^; slices: 39	TBSS; GTS	1	At least 6 months	Yes	Yes
Villemonteix et al. (2015)	sMRI	3.0 T	8	TR/TE: 1960/4.60 ms; FOV: 250x250mm; flip angle: 8°; In-plane voxel size: 0.87 × 0.87 × 1.0mm^3^; slices: 160	VBM; ROI	1	At least 1 year	Yes	Yes
Bouziane et al. (2019)	DTI	3.0 T	8	TR/TE: 8135/94 ms; FOV: 224 × 224 mm; flip angle: not reported; In-plane voxel size: not reported; slices: 60	VBA; ROI	1	18 weeks	Yes	Yes
Walhovd et al. (2020)	sMRI	3.0 T	8	TR/TE: 9.8/4.6 ms; FOV: 256x256x120 mm; flip angle: °; In-plane voxel size: 0.875 × 0.875 × 1.2 mm^3^; slices:	ROI; VBM	1	16 weeks	Yes	Yes
Chang et al. (2021)	sMRI	3.0 T	32	TR/TE: 2000/2.98 ms; FOV: 256 × 256 mm; flip angle: 9°; In-plane voxel size: not reported; slices: not reported	VBM; SVM	1	At least 1 month	Yes	Yes
Chen et al. (2025)	DTI	3.0 T	8	TR/TE: 8135/94 ms; FOV: 224x224x120 mm; flip angle: not reported; In-plane voxel size: not reported; slices: not reported	ROI; CML, Radiomics	2	17 weeks	Yes	Yes
Chang et al. (2025)	sMRI	3.0 T	32	TR/TE: 2000/2.98 ms; FOV: 256 × 256 mm; flip angle: 9°; In-plane voxel size: not reported; slices: not reported	LME; GLM	1	On average 4.86 years	No	Yes

Abbreviations: DTI, diffusion tensor imaging; sMRI, structurel MRI; N/R, not reported; TR/TE, repetition time/echo time; FOV, field of view; FU, follow-up; DO, drop-outs; VBA, voxel based analysis; TBSS, tract-based spatial statistics; GTS, global test statistic; VBM, voxel based morphometry; ROI, region of interest; SVM, support vector machine; CML, conventional machine learning; LME, linear mixed-effects; GLM, general linear model.

Most studies used 3.0 Tesla scanners, indicating generally high-resolution imaging quality. Only one study de Luis-García et al. (2015) used a 1.5 Tesla scanner, which may result in lower signal-to-noise ratio and reduced sensitivity compared to 3.0 T systems. Chen et al. (2025) included two follow-up assessments, whereas all other studies had only one follow-up assessment. There was considerable variation in the total follow-up duration across the studies, ranging from 8 weeks to nearly 5 years. Almost all studies reported drop-outs (DO), except for the studies by Park et al. (2013) and Chang et al. (2025). Still, all studies sufficiently described the follow-up procedures.

We observed substantial heterogeneity in acquisition parameters, including TR/TE, FOV, voxel size, and number of slices. Some studies provided detailed acquisition settings (e.g., Villemonteix et al. (2015)), while others reported incomplete information, with several parameters listed as not reported. This lack of standardization may affect the comparability of findings across studies. Additionally, voxel resolution and slice coverage varied considerably, which can influence the sensitivity to detect structural differences, particularly in small brain regions. Similarly, flip angle was inconsistently reported, and when provided, differed across studies.

### 3.3 Outcomes

Three outcomes were examined: gray matter volume, cortical thickness and white matter integrity. All findings are shown in Table 5.

**Table 5:** Outcomes, findings and limitations of included articles.

Study	Type of MRI/Outcome	Design and subjects	Findings	Limitations
Villemonteix et al. (2015)	sMRI Outcome: GM volume	TD: *n* = 24 Medicated ADHD: *n* = 20 Never-medicated ADHD: *n* = 33	ADHD medicated versus TD children: Medicated children with ADHD exhibited increased GM volume in the middle frontal gyrus and in the precentral gyrus. ADHD medicated versus never-medicated children: the never-medicated children displayed increased GM volume in the middle frontal gyrus, and reduced GM volume in the putamen, the insula, the posterior lobe of the cerebellum and the middle temporal gyrus.	MPH exposure did not exceed over four years Only analyzed one of the many indicators of brain development (GM volume) Lack of longitudinal design



Chang et al. (2021)	sMRI Outcome: GM volume	Drug naive ADHD Good responder: *n* = 63 Poor responder: *n* = 16	Good responders had larger GM volumes in the left putamen cluster and smaller GM volumes in the bilateral precuneus.	Lack of placebo group Excluded participants who were lost to follow-up within 1 month Wide age-range Restricted to striatal and default-mode network masks



Chang et al. (2025)	sMRI Outcome: GM volume	Early exposed <12 years and late exposed. TDC: *n* = 91 ADHD treated: *n* = 89	In children under 12 increased cumulative MPH dosage was associated with increased GM volumes in several frontal areas, such as the right paracentral, caudal middle frontal, superior frontal, lateral orbitofrontal, rostral middle frontal, precentral cortices, left pars opercularis, paracentral, and superior frontal cortices. In children who started MPH after age 12, there were no significant differences, as well as on whole brain exploratory analysis.	Mainly male participants Inclusion of previously treated individuals Study sample was restricted to individuals with higher cognitive functioning and without major psychiatric disorders



Park et al. (2013)	DTI Outcome: FA	Genotype GG: *n* = 25 Genotype CG + CC: *n* = 28 Genotype CC: *n* = 15 Genotype CT + TT: *n* = 38	Effects of norepinephrine genes on DTI Decreased FA values in right postcentral gyrus and right middle frontal cortex.	No control group Small sample size Unequal distribution of gender Less conservative correction for multiple comparisons



de Luis-García et al. (2015)	DTI Outcomes: FA and MD	Healthy Children: *n* = 26 ADHD-nT: *n* = 16 ADHD-uT: *n* = 24	Using TBSS, no differences in FA or MD were detected among medicated, drug-naïve, and healthy control groups. GTS analysis showed that ADHD patients under treatment (ADHD-uT) had lower MD than both healthy controls (HC) and untreated ADHD patients (ADHD-nT) in several major white matter tracts — specifically the inferior longitudinal fasciculus (ILF), uncinate fasciculus (UNC), and sector IV of the corpus callosum (CC). No significant differences in FA	Small sample size two analysis methods, no consensus over optimal way to analyze data No comorbid disorders, precluding generalizability



Bouziane et al. (2019)	DTI Outcomes: FA and MD	MPH treatment: *n* = 25 Placebo: n = 25	No effect in ROI FA analysis VBA analysis revealed significant time-by-medication-by-age interactions: MPH-treated boys showed greater FA (but not MD) increases in left-hemisphere association tracts and the corpus callosum, effects not seen in placebo-treated boys (or adults).	Only boys Nearly all subjects right-handed Limited sample size Single b value



Chen et al. (2025)	DTI Outcome: FA	Good responder: *n* = 17 Poor responder: *n* = 30	Mean FA in the left ATR was lower in the good responders compared to poor responders	Small sample size



Walhovd et al. (2020)	sMRI Outcome: Cortical thickness	MPH treatment: *n* = 25 Placebo: *n* = 23	MPH condition: less cortical thinning in the right medial cortex ROI, but not in placebo condition (or adults)	Small sample size Short follow-up time No significant results between changes in cortical ROIs and changes in symptom severity, thus functional significance uncertain

Abbreviations: DTI, diffusion tensor imaging; sMRI, structural MRI; WMC, white matter connectivity; AP, attentional performance; FA, fractional anisotropy; MD, mean diffusivity; GM, gray matter; ADHD-nT, medication naïve; ADHD-uT, under treatment; TD, typically developing; MPH, methylphenidate; TDC, typically developing children; HC, healthy children; ATR, anterior thalamic radiation.

#### 3.3.1 Gray matter volume

The two sMRI studies (Villemonteix et al., 2015; Chang et al., 2025) indicated GM volume differences associated with MPH: Villemonteix et al. (2015)) compared medication groups cross-sectionally, while Chang et al. (2025) examined cumulative MPH exposurelongitudinally. Villemonteix et al. (2015) reported increased GM volumes in subcortical regions, particularly the putamen, insula, and cerebellum, in medicated children. In frontal cortical regions, findings were mixed, with increased volumes in the middle frontal and precentral gyri compared to typically developing children, but reduced middle frontal gyrus volume compared to never-medicated children. Chang et al. (2025) reported that higher cumulative MPH exposure was associated with increased gray matter volumes across multiple frontal regions, including the superior, middle, orbitofrontal, and precentral cortices, whereas no subcortical effects were observed. These effects were observed only in children who initiated treatment before age 12, with no significant associations in those who started later.

#### 3.3.2 White matter integrity (FA, fractional anisotropy and MD, mean diffusivity)

Across four studies examining white matter structures in children with ADHD (Park et al., 2013; de Luis-García et al., 2015; Bouziane et al., 2019), the assessed outcomes included FA, with only de Luis-García et al. (2015) and Bouziane et al. (2019) reporting both measures (FA and MD) within the same study.

Evidence related to FA was heterogeneous. Park et al. (2013) reported decreased FA in specific regions depending on genetic variation, including the right postcentral gyrus in C allele carriers and the right middle frontal cortex in T allele carriers. In contrast, in the RCT by Bouziane et al. (2019) no significant FA effects in ROI analyses were found, although voxel-based analyses revealed significant time-by-medication-by-age interactions, with greater FA increases in left-hemisphere association tracts and the corpus callosum in MPH-treated boys; these effects were not observed in placebo-treated boys or adults. de Luis-García et al. (2015) found no significant differences in FA or MD using TBSS between medicated ADHD patients, drug-naïve patients, and healthy controls. However, an alternative tract-based analysis (GTS) revealed that medicated patients had lower MD than both groups in key white matter tracts, including the inferior longitudinal fasciculus, uncinate fasciculus, and corpus callosum, while no FA differences were observed, whereas Bouziane et al. (2019) found no MD effects.

#### 3.3.3 Cortical thickness

Only one study examined cortical thickness. Walhovd et al. (2020) investigated regional cortical thickness changes after 16 weeks of MPH treatment in an RCT and found age-dependent, region-specific effects, with increased thickness (or reduced cortical thinning) in the right medial cortex ROI of the three ROIs (left frontal cortex, right medial cortex and right posterior cortex) in children receiving MPH compared with placebo. In contrast, both MPH- and placebo-treated adults showed cortical thinning across regions. Overall, these findings suggest that MPH-related effects on cortical thickness are age-dependent and primarily observable in children.

#### 3.3.4 Baseline prediction studies

In a separate analysis focusing on treatment response, Chang et al. (2021) reported that good responders showed increased gray matter volume in the putamen and reduced volume in the bilateral precuneus compared to poor responders. Using DTI, Chen et al. (2025) found lower FA in the left anterior thalamic radiation in good responders compared to poor responders.

### 3.4 Methodological quality assessment

Table 6a, Table 6b present the methodological quality assessments. Five studies were considered as case series (Park et al., 2013; de Luis-García et al., 2015; Villemonteix et al., 2015; Chang et al., 2021; Chang et al., 2025), and 3 were RCT (Bouziane et al., 2019; Walhovd et al., 2020; Chen et al., 2025).

**Table 6a:** Quality assessment of included articles.

Study	Q1	Q2	Q3	Q4	Q5	Q6	Q7	Q8	Q9	Overall quality^1^
Park et al. (2013)	Yes	Yes	Yes	Yes	Yes	Yes	Yes	Yes	Yes	Good
de Luis-García et al. (2015)	Yes	Yes	N/R	N/R	No	Yes	Yes	Yes	Yes	Fair
Villemonteix et al. (2015)	Yes	Yes	Yes	No	Yes	Yes	Yes	Yes	Yes	Good
Chang et al. (2021)	Yes	Yes	Yes	Yes	No	Yes	Yes	Yes	Yes	Good
Chang et al. (2025)	Yes	Yes	Yes	No	Yes	Yes	Yes	Yes	Yes	Good

Q1: Was the study question or objective clearly stated? Q2: Was the study population clearly and fully described, including a case definition? Q3: Were the cases consecutive? Q4: Were the subjects comparable? Q5: Was the intervention clearly described? Q6: Were the outcome measures clearly defined, valid, reliable, and implemented consistently across all study participants? Q7: Was the length of follow-up adequate? Q8: Were the statistical methods well-described? Q9: Were the results well-described?

1 Overall quality: ‘good’ if 7–9 questions were answered with ‘yes’, ‘fair’ if 4–6 questions were answered with ‘yes’ and ‘poor’ if less than 4 questions were answered with ‘yes’.

**Table 6b:** Quality assessment of RCT.

Study	Q1	Q2	Q3	Q4	Q5	Q6	Q7	Q8	Q9	Q10	Q11	Q12	Q13	Q14	Overall quality^1^
Bouziane et al. (2019)	yes	yes	yes	yes	yes	yes	yes	yes	yes	yes	yes	yes	yes	yes	good
Walhovd et al. (2020)	yes	yes	yes	yes	yes	yes	yes	yes	yes	yes	yes	yes	yes	yes	good
Chen et al. (2025)	yes	yes	yes	yes	yes	yes	yes	yes	yes	yes	no	no	no	yes	moderate/good

Q1. Was the study described as randomized, a randomized trial, a randomized clinical trial, or an RCT?

Q2. Was the method of randomization adequate (i.e., use of randomly generated assignment)?

Q3. Was the treatment allocation concealed (so that assignments could not be predicted)?

Q4. Were study participants and providers blinded to treatment group assignment?

Q5. Were the people assessing the outcomes blinded to the participants' group assignments?

Q6. Were the groups similar at baseline on important characteristics that could affect outcomes (e.g., demographics, risk factors, co-morbid conditions)?

Q7. Was the overall drop-out rate from the study at endpoint 20% or lower of the number allocated to treatment?

Q8. Was the differential drop-out rate (between treatment groups) at endpoint 15 percentage points or lower?

Q9. Was there high adherence to the intervention protocols for each treatment group?

Q10. Were other interventions avoided or similar in the groups (e.g., similar background treatments)?

Q11. Were outcomes assessed using valid and reliable measures, implemented consistently across all study participants?

Q12. Did the authors report that the sample size was sufficiently large to be able to detect a difference in the main outcome between groups with at least 80% power?

Q13. Were outcomes reported or subgroups analyzed prespecified (i.e., identified before analyses were conducted)?

Q14. Were all randomized participants analyzed in the group to which they were originally assigned, i.e., did they use an intention-to-treat analysis?

1 Overall quality: ‘good’ if 10–14 questions were answered with ‘yes’, ‘fair’ if 6–9 questions were answered with ‘yes’ and ‘poor’ if less than 6 questions were answered with ‘yes’.

Overall, the methodological quality of the included case studies was high (Table 6a).

Only the study of de Luis-García et al. (2015) was rated as fair quality due to several unmet or unreported criteria. No studies were classified as poor quality, indicating a generally robust evidence base. Across studies, several domains were consistently well addressed. All studies clearly stated their research objectives (Q1) and described their study populations (Q2). In addition, outcome measures (Q6), follow-up duration (Q7), statistical methods (Q8), and reporting of results (Q9) were almost uniformly adequate, suggesting strong methodological rigor in study execution and reporting. Therefore, overall, the methodology quality can be considered as good, except for the inconsistencies in reporting - particularly regarding intervention details (Q5) and group comparability (Q4) and represent important limitations that should be considered when interpreting the findings.

The methodological quality of the included RCTs are presented in Table 6b. Data of 1 RCT are actually presented in 3 papers (Bouziane et al., 2019; Walhovd et al., 2020; Chen et al., 2025).

## 4 Discussion

MPH treatment characteristics varied widely across studies, with durations ranging from weeks to years (up to 5 years maximum) and dosage often inconsistently or incompletely reported, limiting comparability. The eight included studies also differed substantially in design and in the level of causal inference they support: three used repeated MRI to assess within-subject structural change associated with treatment Bouziane et al. (2019), Walhovd et al. (2020), and Chang et al. (2025); two were cross-sectional comparisons based on medication history de Luis-García et al. (2015) and Villemonteix et al. (2015); and three used baseline neuroimaging to predict subsequent treatment response or clinical outcome rather than to assess MPH-induced structural change Park et al. (2013), Chang et al. (2021), and Chen et al. (2025). These design differences, discussed separately below, mean that most of the included evidence is observational or cross-sectional rather than randomized, which limits causal inference; most studies were nonethelessrated as good methodological quality on the NIH tool (Table 6, and see Section 5.5 for its limitations). Overall, the evidence suggests that MPH- use is associated with both cortical and subcortical brain differences, with more consistent findings in subcortical structures (e.g., the putamen) and more variable findings in the (frontal) cortex. Findings on white matter appear subtle and inconsistent, while findings on cortical thickness are limited to a single study. Age-dependent effects were reported across all outcome measures, although this evidence was based on a limited number of independent studies.

### 4.1 Gray matter volume

Across the three structural MRI studies (Villemonteix et al., 2015; Chang et al., 2021; Chang et al., 2025), subcortical effects—particularly in the putamen—were reported in two studies (Villemonteix et al., 2015; Chang et al., 2021), with increased putamen volume associated with both greater treatment exposure and better treatment response. Consistent with this, Nakao et al. (2011) identified the basal ganglia as the most robust site of ADHD-related structural differences and found that both increasing age and stimulant medication were independently associated with more normalized (i.e., increased) basal ganglia volumes. In contrast, cortical findings were more heterogeneous: Chang et al. (2025) reported that higher cumulative MPH exposure was associated with increased gray matter volume in multiple frontal regions in children who initiated treatment before age 12 only and not thereafter, whereas Villemonteix et al. (2015) reported the opposite pattern, with reduced frontal GM volume observed in medicated children. Neurodevelopmental studies show that brain regions mature at different rates, with particularly prolonged development of the frontal cortex—among the last regions to fully mature—alongside continued structural changes in the striatum throughout childhood and adolescence. This extended developmental trajectory may confer greater sensitivity of these fronto-striatal circuits to treatment effects during earlier stages of development (Andersen and Navalta, 2004). Taken together, the findings suggest that MPH-related brain changes involve both cortical and subcortical regions, with more consistent effects in subcortical structures such as the putamen and more variable, age-dependent effects in frontal cortical areas.

### 4.2 White matter microstructure

We found no strong, converging pattern across the three diffusion MRI studies, with one study reporting decreased FA (Park et al., 2013), one increased FA (Bouziane et al., 2019), and two showing no significant effects in children (de Luis-García et al., 2015; Chen et al., 2025). Although the corpus callosum and fronto-association tracts were implicated, effects varied in direction, suggesting methodological and sample-related influences rather than a robust pattern. Interpretation is somewhat clearer when considering MD alongside FA, as increased FA and decreased MD both indicate improved white matter microstructure. In this context, Bouziane et al. (2019) in the RCT reported increased FA in left-hemisphere association tracts in MPH-treated children (but not adults or placebo groups), while de Luis-García et al. (2015) found reduced MD in major tracts, together suggesting a potential positive effect of MPH on white matter organization. This finding is broadly consistent with prior evidence showing increased white matter volume in stimulant-medicated children (Castellanos et al., 2002), as well as an association between greater cumulative stimulant exposure and lower MD in the orbitofrontal–striatal pathway (Schweren et al., 2016). Schweren et al. (2016), like Shaw et al. (2009) and van der Pal et al. (2024) below, is cited here as supporting evidence rather than as an included study, as it examined stimulant treatment history broadly rather than MPH specifically, consistent with this review's a priori restriction to MPH studies for comparability (Section 2.2.1). It also aligns with animal studies reporting increases in FA in adolescent, but not adult, rats following stimulant exposure (van der Marel et al., 2014), potentially linked to enhanced expression of axon-related genes induced by early MPH treatment (Adriani et al., 2006). Overall, the combined evidence tentatively suggests that early MPH treatment may enhance white matter maturation and microstructural organization in children with ADHD, with effects appearing more pronounced during developmental periods than in adulthood.

### 4.3 Cortical thickness

Only one study, a RCT (ePOD-MPH; Walhovd et al. (2020)) examined cortical thickness and found that MPH increased cortical thickness in the right medial cortex ROI in children after four months, an effect not observed in the placebo group, nor adults. This is in line with Shaw et al. (2009), who reported similar effects of stimulant treatment (not specifically MPH) in the same ROI and additional regions, suggesting that stimulants may modulate typical cortical thinning trajectories. However, a four-year follow-up of the ePOD-MPH RCT by van der Pal et al. (2024) found no association between stimulant use and cortical thickness in this ROI, indicating that these effects may be transient. Shaw et al. (2009) and van der Pal et al. (2024) were not among the eight studies appraised in this review, as both examined broader stimulant medication use rather than MPH specifically, consistent with this review's a priori restriction to MPH studies for comparability (Section 2.2.1). In addition, no cortical thickness effects were observed in adults after four months, unlike in children Walhovd et al. (2020)). Overall, these findings suggest that MPH-related cortical effects are age-dependent and possibly short-lived.

### 4.4 Treatment response

Two MRI studies (Chang et al., 2021; Chen et al., 2025) examined long-term MPH response and found that treatment efficacy is associated with distributed differences across both gray and white matter systems. Good responders showed increased gray matter volume in subcortical regions, including the putamen, insula, and cerebellum, along with reduced precuneus volume (Chang et al., 2021), and also exhibited lower FA in the left anterior thalamic radiation (Chen et al., 2025). Together, these findings suggest that MPH response is linked to a specific pattern of neurodevelopmental organization involving both subcortical structure and fronto-parietal white matter pathways, rather than isolated regional abnormalities. This supports a network-level view of treatment response, where individual differences in brain organization may help explain variability in clinical outcome.

### 4.5 Strength and limitations

The evidence base of this systematic review is limited by substantial heterogeneity in study design, sample characteristics, and MPH treatment protocols, which reduces comparability and limits generalizability. Additional constraints include reliance on a single RCT, (reported in three publications), exclusion of comorbid conditions, and inclusion of mixed age groups, all of which further restrict clinical interpretation. Despite these limitations, most studies were rated as good methodological quality on the NIH quality assessment tools for case series and RCTs, although incomplete reporting of interventions and occasional group imbalances may introduce bias. However, the NIH tool does not assess randomization, allocation concealment, blinding, attrition, or confounding, and therefore does not fully capture risk of bias for either the randomized trial reports or the observational studies included here; a formal re-appraisal using RoB 2 for the randomized trial reports and ROBINS-I (or the Newcastle-Ottawa Scale) for the observational studies is warranted before the strength of this evidence base can be considered fully established. Furthermore, three of the eight included studies—Bouziane et al. (2019), Walhovd et al. (2020), and Chen et al. (2025)—are not independent but are three reports derived from the same ePOD-MPH randomized controlled trial, sharing a 2011–2015 recruitment window and an overlapping sample of approximately 50 boys. As a result, the effective number of independent datasets underlying this review is therefore closer to five or six than eight, and this should be borne in mind when interpreting the strength of the evidence base. Two authors of this review (LR, ZvdP) are co-authors of these three studies (see Declaration of Competing Interest). Another limitation is that this review was not prospectively registered (e.g., in PROSPERO), no formal a priori protocol was developed, and that only two databases (MEDLINE and EMBASE) were searched which may have resulted in missed studies. However, comprehensive search strategies and routine screening of reference lists and forward citations of included studies may have reduced this risk. We therefore consider the potential impact on the overall findings to be limited. Title/abstract screening was performed by a single reviewer (DS), with adjudication by a second reviewer (SB) only in cases of uncertainty; no formal inter-rater agreement statistics were calculated. Two included studies, Chang et al. (2021) and Chang et al. (2025), have mean sample ages of 17.6 (SD 10.1) and 17.59 (SD 8.00) years respectively, indicating that a proportion of participants were young adults or transitional-age youth rather than children; indicating a child-versus-adult contrast rather than a developmental gradient within childhood. Overall, while methodological rigor is relatively strong, on several dimensions, the risk-of-bias limitations, design heterogeneity, and reporting gaps described above should be considered when interpreting the findings. A key strength of this review is its focus on MPH only, excluding other stimulants with different pharmacological mechanisms, thereby improving comparability across studies. This is important because MPH primarily acts as a dopamine reuptake inhibitor, whereas amphetamine derivatives also promote dopamine release, reflecting distinct pharmacological profiles (Faraone, 2018) and have been associated with neurotoxic effects in preclinical studies (Ricaurte et al., 2005).

### 4.6 Future outlook

Future research should prioritize larger, well-powered longitudinal and meta-analytic studies to better characterize the developmental effects of MPH on brain structure. Clinical relevance would be enhanced by including children with comorbidities and by standardizing key methodological factors such as MRI protocols, treatment duration, and dosage reporting. In addition, this review highlights the importance of accounting for moderating factors such as age and developmental stage to better explain variability in treatment effects and response. Finally, longer follow-up periods—beyond five years—are needed to better reflect real-world MPH exposure, as many children remain on treatment for extended durations. The longest prospective follow-up to date, the Multimodal Treatment Study of ADHD (MTA), extending up to eight years, showed that initial between-group differences diminish over time, suggesting that early behavioral benefits are not maintained at long-term follow-up (Sibley et al., 2024). However, it remains unclear whether similar trajectories apply to brain structural outcomes, as the here reviewed neuroimaging follow-up studies were limited to approximately five years, warranting further investigation.

## 5 Conclusion

Overall, MPH treatment is associated with increased gray matter in subcortical regions, particularly the putamen, with more variable effects in frontal cortex. White matter findings are inconsistent, and may modestly enhance white matter microstructural organization in children with ADHD. Evidence on cortical thickness is limited and suggests short-term, possibly non-persistent effects. Across all outcomes, age appeared to be a key moderating factor. Future studies should use larger, standardized, longitudinal designs that better capture developmental effects of treatment.

## Declaration of generative AI and AI-assisted technologies in the manuscript preparation process

During the preparation of this work the authors used ChatGPT for final proofreading of the text. After using this tool/service, the authors reviewed and edited the content as needed and takes full responsibility for the content of the published article.

## CRediT authorship contribution statement

**Liesbeth Reneman:** Writing – review & editing, Writing – original draft, Supervision, Project administration, Methodology, Data curation, Conceptualization. **Zarah van der Pal:** Writing – review & editing, Methodology, Investigation, Conceptualization. **Daniëlle de Smidt:** Writing – original draft, Visualization, Validation, Investigation, Formal analysis, Data curation. **Shandra Bipat:** Writing – review & editing, Writing – original draft, Validation, Supervision, Project administration, Methodology, Formal analysis, Data curation, Conceptualization.

## Role of the funder/sponsor

The funding sources had no role in the design and conduct of the study;collection, management, analysis, and interpretation of the data; preparation, review, or approval of the manuscript; and decision to submit the manuscript for publication.

## Funding

This work is supported by a grant from Kiddy Goodpills (2019).

## Declaration of competing interest

The authors declare that they have no known competing financial interests or personal relationships that could have appeared to influence the work reported in this paper. Two of the authors of this review (LR, ZvdP) are co-authors of three of the eight included primary studies (Bouziane et al. 2019, Walhovd et al. 2020, and Chen et al. 2024). However, the authors who performed the primary data-extraction extraction were not involved in the RCT.

## Data Availability

No data was used for the research described in the article.

## Appendix A Supplementary data

Supplementary material: Search Strategy
