# Supplementary material for: Methylphenidate-associated brain structural changes and neuroimaging predictors of treatment response in youth with ADHD: a systematic review
Source: Neuroimage Clin. 2026 Nov 3;52:104085. doi: 10.1016/j.nicl.2026.104085 (PMC13769477; doi:10.1016/j.nicl.2026.104085)
Supplement: Supplementary file 1 [file mmc1.docx]

Supplement 1. Search strategy

| MEDLINE (search until 1 September 2025) | Search terms | Hits |
| --- | --- | --- |
| #1 | attention deficit disorder with hyperactivity | 51399 |
| #2 | methylphenidate | 10743 |
| #3 | magnetic resonance imaging | 752519 |
| #4 | Brain | 2530530 |
| #1 AND #2 AND #3 AND #4 |  | 179 |
|  |  |  |
| EMBASE (search 1 until September 2025) |  |  |
| #1 | 'attention deficit disorder with hyperactivity'/exp | 89582 |
| #2 | 'methylphenidate'/exp | 23126 |
| #3 | 'magnetic resonance imaging'/exp | 1411156 |
| #4 | 'brain'/exp | 1468914 |
| #1 AND #2 AND #3 AND #4 | LIMIT HUMAN AND NOT MEDLINE | 328 |
